# Improvement in cognitive impairment following the successful treatment of endogenous Cushing’s syndrome-a case report and literature review

**DOI:** 10.1186/s12902-019-0401-4

**Published:** 2019-06-28

**Authors:** Malgorzata Monika Brzozowska, Sacha Kepreotis, Fiona Tsang, Sully Xiomara Fuentes- Patarroyo

**Affiliations:** 1 Endocrinology Department, Sutherland Hospital, Sydney, NSW Australia; 2 Faculty of Medicine, University of New South Wales, Kensington, NSW Australia; 3 Garvan institute of Medical Research, Darlinghurst, NSW Australia

**Keywords:** Cushing’s syndrome, Cognitive deficit, Functional decline, Microvascular ischaemic changes, Transsphenoidal excision, Pituitary microadenoma

## Abstract

**Background:**

Endogenous Cushing’s syndrome, a rare endocrine disorder, characterised by chronic cortisol hypersecretion, results in neuropsychiatric disturbances and in cognitive deficits, which are only partially reversible after the biochemical remission of the disease.

**Case presentation:**

We report a case of a woman with a profound cognitive deficit and a gradual functional decline caused by Cushing’s disease of at least 10 years duration. The neurosurgical resection of her 2 mm adrenocorticotropic hormone (ACTH) secreting pituitary microadenoma resulted in a successful resolution of the patient’s hypercortisolism and a significant recovery of her neurocognitive function. The patient’s progress was evaluated using serial clinical observations, functional assessments, Mini-Mental Status exams and through the formal neuropsychological report. Furthermore, the patient’s recovery of her neurocognitive function was reflected by a sustained improvement in the patient’s specific structural brain abnormalities on radiological imaging.

**Conclusions:**

This report illustrates the importance of early detection and treatment of Cushing’s syndrome in order to prevent neurocognitive impairment and neuropsychiatric disorders which are associated with an endogenous cortisol hypersecretion. The long term adverse effects of severe hypercortisolaemia on brain function and the pathophysiological mechanisms responsible for the structural and functional changes in brain anatomy due to glucocorticoid excess are reviewed.

## Background

Cushing’s disease (CD), a rare disorder with an incidence of 1.2–1.7 per million population per year [1], is caused by a disruption of the hypothalamus-pituitary-adrenal (HPA) axis with a pathological increase in circulating cortisol secondary to excessive adrenocorticotropic hormone (ACTH) production. The cortisol excess is associated with increased mortality due to serious comorbidities including systemic arterial hypertension, insulin resistance with diabetes mellitus, hypercoagulable state and decreased bone mineral density [2, 3]. Additionally CD results in neuropsychiatric disturbances with major depression and generalized anxiety disorders being diagnosed in 54–81% of patients with CD [4] well as in neurocognitive deficits with memory impairments, deficits in visual and spatial information, reasoning and verbal intellectual skills and memory recall [5, 6]. Sustained exposure to glucocorticoid excess can induce hippocampal atrophy and cerebral remodelling via numerous pathological mechanisms including decreased glucose utilisation, disruptions in neuronal homeostasis, as well as aberrations in neurotransmitter signalling [5] [7]. The limited studies evaluated the long-term effects of chronic overexposure to cortisol, such as in CD, on the brain. The majority of these studies pointed to the long standing deficits affecting verbal skills and visual memory despite biochemical remission [8].

We report a case of a woman with a profound cognitive deficit and a gradual functional decline caused by the CD of at least 10 years duration. The neurosurgical resection of her 2 mm ACTH secreting pituitary microadenoma resulted in a successful reversal of patient’s hypercortisolism and a significant recovery of her cognitive function, evident at 3.5 years after her neurosurgery.

### Case presentation

A 59-year-old woman, previously independent in activities of daily living and self-employed in the family business, presented with a progressive, over the period of 10 years, decline in cognitive function manifested as increasing social withdrawal, inattentiveness followed by progressive memory impairment, inappropriate behaviour, urinary incontinence and problems with balance. Furthermore she had episodes of an emotional lability, alternating between depressive symptoms with psychomotor retardation and agitation with paranoid ideation, insomnia and confusion. Patient’s presentation was complicated by a spontaneous (8 mm) subdural haematoma treated with craniotomy and evacuation in year 2014 as well as by a single seizure during the perioperative period with a subsequent pulmonary embolism requiring temporary use of anticoagulation. Despite surgical evacuation of her subdural haematoma, patient’s cognitive status continued to deteriorate due to superimposed episodes of delirium requiring multiple, 2nd monthly hospitalisations. Comprehensive geriatric assessment, performed in between hospitalisations, confirmed an impairment of the patient’s executive function with impaired reasoning and problem solving skills. Her Mini-Mental state examination (MMSE), a brief (5–10 min) mental status questionnaire, assessing attention, orientation, memory, language and visuospatial copying, revealed a score of 24/30, suggestive of cognitive decline. 3 months prior to the neurosurgery her executive function severely declined to the point of requiring fulltime assistance with all daily living activities including her personal care and the need for regular antipsychotics and antidepressants with haloperidol, mirtazapine and levetiracetam. On her preoperative assessment (Barthel Index of activities of daily living, total score range from 0 to 20) the patient scored only 7 points, indicative of her severe limitations in activities of daily living [9].

The patient’s past medical history included hypertension, type 2 diabetes mellitus, thyrotoxicosis successfully resolved with I-131 treatment, osteoporosis with L5 vertebral compression fracture, recurrent urinary tract infections requiring supra-pubic catheterisation and hyperandrogenism with an onset after 40 years of age.

### Presentation and investigations

The patient had mild phenotypic Cushingoid features including round face, central obesity, skin thinning and buffalo hump, mild facial hirsutism, kyphosis without vertebral tenderness and mild proximal myopathy.

The initial investigations revealed raised serum cortisol at 768 nmol/L (Reference range (RR) 155–599) with normal ACTH of 35.1 pg/mL (RR between 7.2 to 62.3), Table 1. Her 24-h urine free cortisol was raised at 413 nM/24 h (RR < 166) with a 24-h urine creatinine of 10 mmol/24 h. An overnight 1 mg dexamethasone suppression test showed non-suppressed serum cortisol of 122 nmol/L [10] with ACTH of 18.5 pg/mL. The results of inferior petrosal vein sampling were consistent with Cushing syndrome of pituitary origin, Table 1.

**Table 1:** Results of initial laboratory tests

Parameters	Parameter Value	Reference interval
Sodium (mmol/L)	138	135–145
Potassium (mmol/L)	5.0	3.6–5.1
Bicarbonate (mmol/L)	24	22–32
eGFR (mL/min) > 90	77	➢ 90
Fasting glucose (mmol/L)	9.7*	3.0–7.8
HbA1c (%) (mmol/mol)	**7.9**	< 6 (42)
Albumin (g/L)	37	33–48
Alanine aminotransferase (U/L)	18	< 45
Aspartate aminotransferase (U/L)	18	< 45
Alkaline phosphatase (U/L)	66	38–126
γ-Glutamyl transferase (U/L)	22	0–30
Bilirubin (umol/L)	6	0–25
Free T4 (pmol/L)	17.3	12–22
Free T3 (pmol/L)	3.5	3.1–6.8
TSH (mIU/L)	1.5	0.27–4.2
Testosterone (nmol/L)	**3.3**	0–1.7
Androgen free index (%)	**12**	0–4.6
DHEAS (umol/L)	**12.9**	2.1–8.7
LH (mIU/ml)	**11.3**	> 30
FSH (mIU/L)	81.7	20–90
Prolactin (mU/L)	**662**	102–496
IGF1 (nmol/L)	25	3.8–29.8
Haemoglobin (g/L)	143	115–165
Platelets (× 109/L)	295	150–450
Confirmation of Cushing syndrome		
0800 cortisol (nmol/L)	**768**	155–599
ACTH (pg/mL)	35.1	7.2–63.3
24 h urinary free cortisol (nmol/day)	**413**	(< 166)
Urine creatinine (24 h)	10	(6.0–18)
Cortisol post 1 mg dexamethasone suppression test (nmol/L)	**122**	155–599
ACTH post 1 mg dexamethasone suppression test (pg/mL)	**18.5**	7.2–63.3
Cortisol post 8 mg dexamethasone suppression test (nmol/L)	109	155–599
ACTH post 8 mg dexamethasone suppression test (pg/mL)	12.6	7.2–63.3
BIPSS: Central-to-peripheral ACTH maximal ratio (baseline)	**17.4**	≥2
BIPSS: Central-to-peripheral ACTH maximal ratio (5 min after CRH stimulation)	**9.7**	≥3

* *ACTH* adrenocorticotropic hormone, *BIPSS* Bilateral inferior petrosal sinus sampling, *DHEAS* dehydroepiandrosterone sulfate, *eGFR* estimated glomerular filtration rate, *FSH* follicle-stimulating hormone, *LH* luteinising hormone, *IGF1* Insulin-like growth factor, *TSH* thyroid-stimulating hormone; bolded results are outside of the reference range

The patient had negative human immunodeficiency virus (HIV) and syphilis serology.

**Fig. 1:**
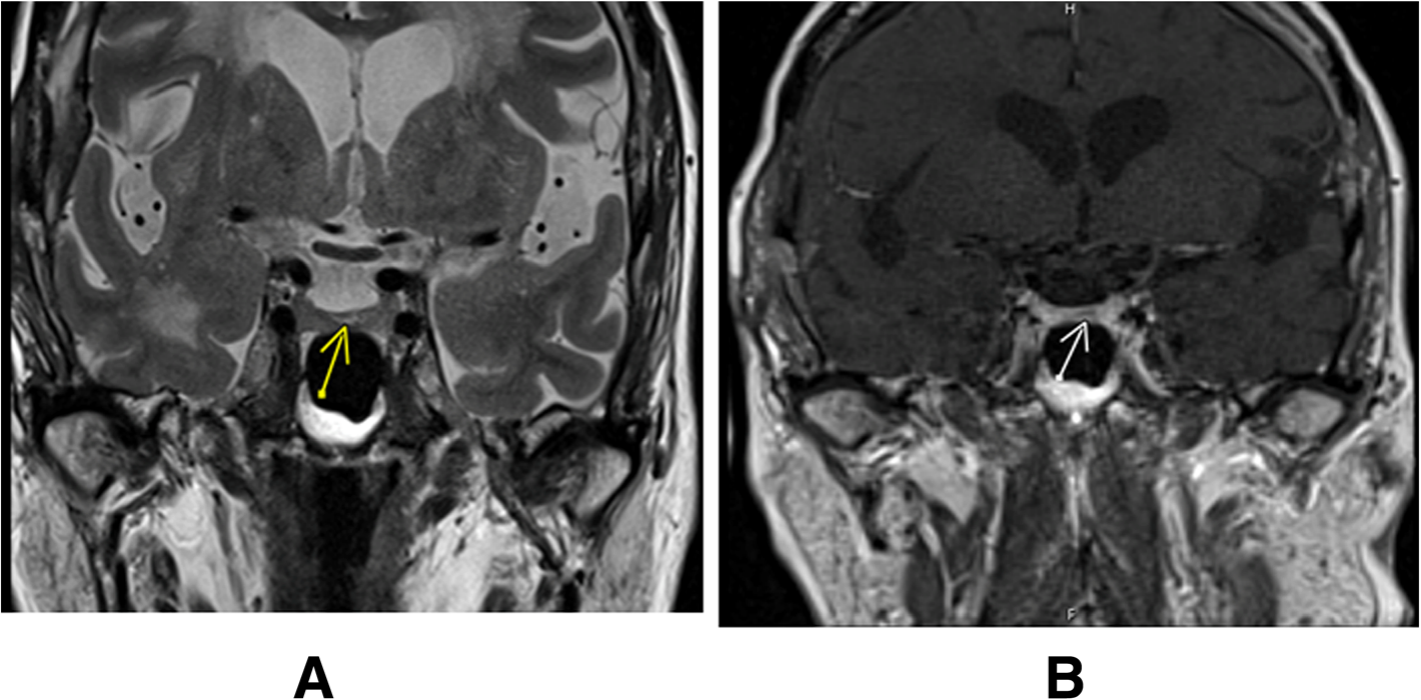
Preoperative MRI showing small pituitary microadenoma. Legend: Coronal MRI views show altered signal intensity within adenohypophysis T2 weighted sequence (Panel **a**), a region of delayed enhancement on the dynamic sequences (Panel **b**)

The patient’s initial magnetic resonance imaging (MRI) of the brain showed extensive white matter disease, in excess of expected for patient’s age, involving both hemispheres, consistent with severe chronic small vessel ischaemia and moderate cerebral atrophy. A subsequent MRI with targeted pituitary views showed a 2 mm pituitary adenoma in the mid portion of the pituitary gland, Fig. 1 The prolonged EEG revealed no evidence of any epileptiform activity.

### Treatment

A stereotactic endoscopic transphenoidal excision of the pituitary microadenoma was performed (11 November 2015). Histopathological analysis of the tumour revealed positive immunostaining for ACTH, equivocal immunostaining for prolactin with negative immunostaining for GH, FSH, LH, TSH and p53. The proliferation index assessed by Ki-67 was less than 1%.

### Outcome and follow up

The post-operative MRI brain showed no evidence of residual pituitary disease. Her serum cortisol level, which was measured on the fourth postoperative day, has decreased to 108 nmol/L, together with ACTH level of 3.6 pg/mL. The patient was commenced on a supraphysiological prednisolone dose of 10 mg/day to reduce her postoperative glucocorticoid withdrawal syndrome. The choice of glucocorticoid replacement therapy was influenced by the patient’s preference for once-daily glucocorticoid dosing. The patient had an uneventful post-operative recovery with a weaning regime of prednisolone up to 12 months post neurosurgery.

As the patient’s prolactin level remained raised on repeated measurements, despite gradual withdrawal of antipsychotic medications, the patient was commenced on cabergoline for a period of 6 months (0.25 mg a week) with a subsequent decline in serum prolactin from 758 mU/L to 299 mU/L (2016). Her prolactin levels remain within the reference range after cabergoline cessation. The patient remains in remission from her Cushing syndrome with the last measured 24 h urinary free cortisol (13 February 2019) being 91 nmol/ 24 h.

At 5 weeks post transphenoidal resection the patient was noticeably more engaging, spontaneous and alert than previously, although still exhibited difficulty with higher order thinking and memory recall. At 5 months the patient continued to display excellent clinical recovery, demonstrating appropriate and spontaneous conversation together with an improvement in short term memory recall and restoration of functional performance with household tasks and she no longer required antipsychotic medications. By 17 months post-surgery, the patient’s cognitive function has further improved with complete reinstatement of her articulation, language and verbal fluency. The patient regained full functional independence with activities of daily living and furthermore, she was able to reassume her duties helping with the family business finances, an improvement reflected by increased postoperative Barthel Index scores to 18 out of maximum 20 points (year 2017). Her mood has improved and therefore she no longer required treatment with antidepressants. The recent (year 2018) MMSE examination, revealed a total score of 30/30. A more comprehensive neuropsychological assessment, performed 3.5 years after the successful neurosurgery (April 2019), revealed deficits in multiple cognitive domains including executive function with difficulty planning and organising, short term memory impairment for visually presented material and deficit in language with poor performance on confrontational word naming ability. The overall assessment was consistent with a mild cognitive impairment in a 63 year old woman, which was related to her previous CD.

**Fig. 2:**
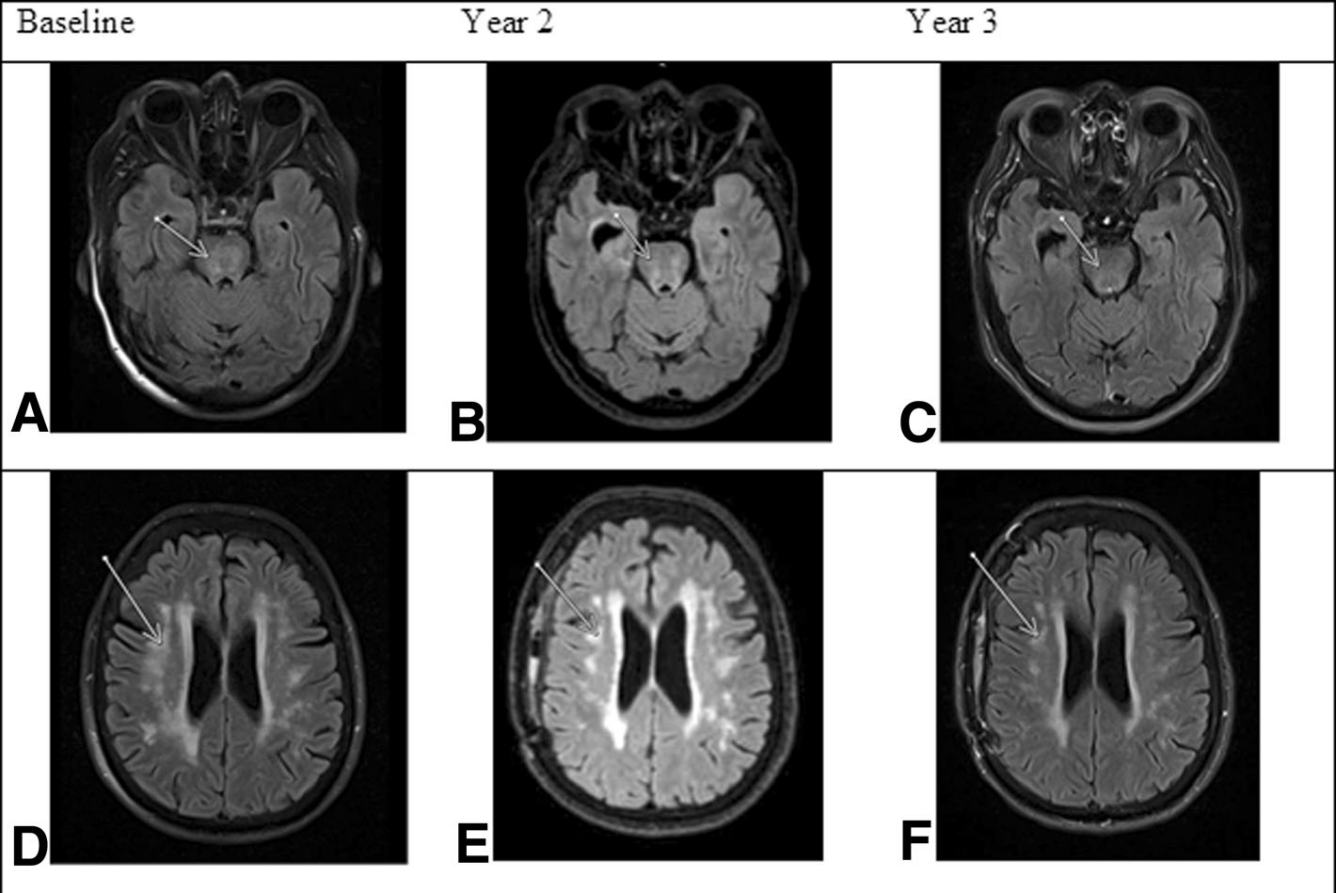
Resolution of small vessel ischemia following the pituitary adenoma resection. Legend: Imaging studies obtained before and after treatment. Axial and sagittal FLAIR MRI images show the progressive resolution of microvascular ischaemic changes in the brainstem and cerebral hemispheres over time. Baseline (Panels **a** and **d**), at 2 years (Panels **b** and **e**) and at 3 years after resection of pituitary adenoma (Panels **c** and **f**)

Progress MRI of the brain revealed significant reduction in the extent of the cerebral hemispheric and pontine “microvascular ischaemic changes”, Fig. 2

Notably the patient experienced resolution of her metabolic abnormalities with improved blood pressure control and resolution of her diabetes with subsequent cessation of her antihypertensive and glucose lowering therapy.

## Discussion

Metabolic disorders are uncommon but potentially reversible causes of progressive dementia. The potentially reversible causes of cognitive decline due to dementia account for less than 10% of dementia presentations. Furthermore less than 1% of these dementia cases have been fully reversible with treatment, usually improving within the first 2 years of diagnosis [11].

We present the case of the patient with CS of at least 10 years duration who initially presented with severe cognitive impairment, affecting multiple functional domains, depression, memory deficits, verbal and language disturbance. The severe cognitive impairment resulted in patient requiring complete assistance with daily activities with a meaningful psychiatric involvement and regular treatment with the antipsychotic agents. The successful neurosurgical excision of her 2 mm pituitary adenoma resulted in dramatic reversibility of her cognitive impairment and her depressive disorder. Furthermore, there was a significant reversibility of the cerebral hemispheric and pontine “microvascular ischaemic changes”and reduction in the ventricular size together with an improved scoring in assessments of her cognitive function and physical disability.

In the present case, due to the severity of patient’s illness, her initial and postoperative cognitive assessments included serial clinical observations, mental status questionnaires together with functional assessments of her activities of daily living. Notably, the MMSE scoring may significantly underestimate cognitive impairment in a large proportion of individuals including patients affected by a mood disorder [12, 13]. As our patient’s cognitive dysfunction has remarkably improved during her first postoperative 6 months, the patient was reluctant to undergo early neuropsychological testing to examine more discrete aspects of her cognitive functioning. The results of her detailed neuropsychological tests, performed at 3 years after her CS treatment, although indicative of the residual functioning impairment and deficits in her visuospatial short term memory, point to a significant recovery of her brain cognitive function and ongoing remission from her CD.

Endogenous glucocorticoid excess in CS has profound effects on the human brain. Patients with active CS demonstrate cognitive impairments that may affect their concentration, learning and memory as well as cause mood disorders including depression, euphoria and anxiety [14]. Compared to healthy controls, patients treated for CD demonstrate impaired verbal functions, in particular learning [15–20], impaired quality of life [21], a higher prevalence of psychopathology (e.g. affective disorders especially major depression and apathy) and maladaptive personality traits [20]. Interestingly, the presence of psychopathology is significantly associated with older age, female gender, higher pre-treatment 24-h urinary cortisol levels, a more severe clinical condition and an absence of pituitary adenoma [22].

The prolonged exposure to high levels of endogenous cortisol may have deleterious, long-lasting effects on the hypothalamic-pituitary-adrenal (HPA)-axis with the irreversible structural brain abnormalities and overall brain atrophy. The review of past studies of patients with active Cushing’s syndrome consistently points to profound structural brain abnormalities with cortical atrophy, smaller hippocampal volume [23] and increased third ventricle and bicaudate diameters [24]. The glucocorticoid excess may affect important centres for learning and memory resulting in widespread changes in white matter, hippocampus and medial temporal lobe (MTL) encompassing amygdala and the anterior cingulate cortex (ACC) [24–28]. A recent study demonstrated smaller grey matter volumes of the bilateral cerebellar hemispheres in patients with active CS compared with controls [29]. Furthermore, children with CS were found to have smaller cerebral volumes, larger ventricles and smaller amygdala in comparison with healthy controls with a subsequent cognitive decline despite reversal of brain atrophy 1 year after resolution of CS [17]. The study of adolescents with chronic endogenous hypersecretion of cortisol, examined by functional magnetic resonance imaging (fMRI), revealed the functional alterations in amygdala and hippocampus [30].

The use of the new imaging techniques such as magnetic resonance imaging (MRI) and^18^F-fluorodeoxyglucose positron emission tomography, further advance our understanding of functional deficits in people with CS. The recent meta-analysis of 19 studies with 339 patients examined brain characteristics in patients with CS (active and in remission) using structural (*N* = 14) and functional (*N* = 5) magnetic resonance imaging [8]. The regional brain neurochemistry was explored in three studies with proton magnetic resonance spectroscopy (H-MRS) while two studies investigated patients’ brain activity with functional MRI (fMRI) [8]. Additionally, radiological findings from nine studies were linked with an assessment of patients’ cognitive function. Patients with active CS, in comparison to healthy controls, were affected by cerebral atrophy with smaller hippocampal and cerebellar cortex volumes as well as by reduced amygdala with coexistent alterations in neurochemical concentrations and brain functional activity [17, 24, 28, 29, 31]. The brain neuronal dysfunction resulted in altered brain metabolites with decrease in the choline to creatinine ratio (Cho/Cr) in frontal (by 24%) and thalamic (by 17%) areas as compared to healthy subjects [31]. Two fMRI studies pointed to changes in brain structures responsible for an emotional processing [30, 32]. Interestingly, the study, which examined brain function in adolescents, noted their preserved affect and cognition despite functional alterations in amygdala and hippocampus suggesting that phenotypic responses to glucocorticoid excess vary depending on patients’ age and stage of their brain maturation [31].

The study of 92 patients with CS, examined by ^18^F-fluorodeoxyglucose positron emission tomography confirmed a link between impaired brain metabolism in several brain regions such as hippocampus, amygdala, cerebellum, frontal and occipital cortex and elevated cortisol levels [33]. Moreover, the results from this study pointed to the potential for the recovery of brain function after cortisol correction with the presence of transient hypometabolism instead of permanent neuronal loss in brain areas involved in the regulation and action of glucocorticoids. The direct evidence linking prolonged endogenous hypercortisolism with subsequent neuronal dysfunction resulting in mood disturbances, in particular anxiety, comes from measurements of altered brain metabolites by proton magnetic resonance spectroscopy (^1^H-MRS) [34]. Taken together, these findings imply that glucocorticoid excess influences the metabolic activity in brain regions with abundant corticosteroid receptors and thus will facilitate exploration of the mechanisms of cognitive disorders in patients with abnormal glucocorticoid levels.

There is a paucity of literature data reporting brain characteristics in CS patients who sustained long term remission from their disease. Furthermore the interpretations of results from these studies is complicated by their different designs, heterogeneity of examined samples, different imaging modalities and duration of follow up ranging from 3.4 to 11.9 years [8]. In the majority of these studies the observed loss of brain volume has only partly recovered after biochemical cure with widespread reductions in white matter integrity [7, 25] with partial increase in hippocampal volume which correlated significantly with the reduced urinary free cortisol [35]. The limbic structures, especially the hippocampus, express the high levels of the mineralocorticoid (MR) and glucocorticoid receptors (GR) with high sensitivity to glucocorticosteroids [36]. The metabolism of glucocorticoids is regulated by enzymes 11-beta-hydroxysteroid dehydrogenases (11b-HSDs) [37]. Polymorphisms in the (GR) gene may contribute considerably to the diverse individual responses to the glucocorticoids [38]. The variability in the expression of GR in pituitary and adrenocortical cells may alter the sensitivity of the hypothalamus-pituitary-adrenal (HPA) axis with subsequent differences in body composition and metabolic factors [38]. The ER22/23EK polymorphism of GR gene has been associated with partial form of GC resistance [39] while BclI and N363S polymorphisms have been linked with higher GC sensitivity [40, 41].GR polymorphisms in BclI and N363S as well in 1β-HSD1 may increase individual predisposition to mood disorder including depression [42, 43]. Interestingly, recent reports have cautiously linked genetic variants of GR with the degree of postoperative neuropsychiatric disorders in CS patients. In a previous study, the polymorphisms in 11 *훽* -HSD type 1 and NR3C1 Bcl1 genes influenced the severity of cognitive impairments in processing and reading speed, auditory attention and working memory together with fatigue [44]. The molecular mechanisms of these observations remain largely unexplained; therefore, much more must be learned about the HPA axis and its regulation.

The previous studies have shown consistently that cognitive deficits in people in remission from CS have improved with change in hippocampal size [15, 45, 46] with substantial improvements in clinical and behavioural outcomes of treated patients during their first postoperative year [47]. Following intervention to reduce glucocorticoids, CD patients show nearly 10% hippocampal volume increases after 1.5 to 2 year [35]. The extent of anatomical abnormalities on MRI findings correlated to changes in patients’ behaviour and mood [48], improved learning as assessed by neuropsychological test performances [45] and altered decision making [49]. The degree of cognitive impairment and cerebral atrophy were associated with patients’ age, age at the time of diagnosis, duration of symptoms [28] and with changes in selective biochemical parameters, in particular with examined cortisol levels [23].

Therefore, the structural and neurochemical abnormalities in both grey- and white matter are not completely reversible at long-term remission and are accompanied by psychological symptoms and impairments in cognitive functioning [7, 8]. In line with our report, the study of thirty-three patients with CS (11 active, 22 cured) noted that verbal and visual memory was worse in CS patients than controls, even after biochemical cure [18]. The recent functional magnetic resonance imaging of 19 women with CS in remission of median 7 years (IQR 6–10) duration identified decreased functional brain responses in the prefrontal cortex and hippocampus [50]. The episodic and working memory testing linked these memory deficits with changes in the prefrontal cortex, a key region for cognitive function, as well as with decreased functional brain response in the hippocampus during episodic memory encoding [50].

Interestingly, the limited studies have shown negative impact of raised prolactin on a range of neurocognitive domains encompassing memory and executive function, anxiety and depressive behaviour as well as in regulation of stress responses [51–55]. Furthermore, recent study demonstrated that treatment of hyperprolactinemia with cabergoline resulted in a cognitive enhancement in patients with prolactin-secreting pituitary adenomas [56]**.** Mechanisms responsible for the cognitive deficits in patients affected by hyperprolactinaemia have not been fully explored. The limited in vivo or ex vivo animal studies linked hyperprolactinemia with the modulation of non-spatial cognitive tasks [54]. The recent cross-sectional clinical study of female patients with prolactinomas, examined with MRI scans, demonstrated decrease in grey matter volume (GMV) in the left hippocampus and prefrontal cortex. These observed structural brain abnormalities were linked to the deficits in verbal memory and executive function on neuropsychological testing [57].

The treatment of CS in our patient resulted in an improvement in patient’s hyperandrogenism with the marked reduction in testosterone and DHEAS levels. The androgens play numerous, important roles in neurocognitive function and mood stability with the powerful effects on executive functioning. Androgens act through binding to intracellular androgen receptor proteins, localised in the multiple brain regions, with the high concentrations in hypothalamic and limbic regions [58, 59]. Interestingly, the studies examining the effects of testosterone on cognition have produced conflicting results. The testosterone has been shown to exert sex-specific effects with both positive and negative influences on cognitive performance. The Dehydroepiandrosterone (DHEA) and its sulfate bound form (DHEAS) have neuroprotective effects and antiglucocorticoid activity [60]. The low androgen levels have been associated with reduced cognitive function, poorer general sense of well-being, impairment of sexual function and depressive symptoms in elderly age [61–63]. Low androgen levels have been reported as a risk factor for development of Alzheimer disease in men [64] while for women, their free testosterone level was negatively associated with verbal fluency, semantic memory, and episodic memory [65]. Importantly, testosterone therapy improved the physical and emotional well-being as well as sexual function of postmenopausal women [66–68]. Conversely, in the group of postmenopausal women, higher testosterone concentration was associated with lower scores for verbal and visual memory, processing and psychomotor speed, executive functions, complex attention and cognitive flexibility [69].

## Conclusions

In summary, we report a case of a woman with a profound cognitive deficit and a functional decline, who after the successful cure of her CS, experienced a significant recovery of her neurocognitive function, reflected by changes in structural brain abnormalities on her MRI imaging. Her presentation, although illustrative of acute and long-term detrimental effects of hypercortisolaemia on brain function, at the same time indicates the potential for the significant recovery of the neurocognitive impairment in patients affected by the endogenous glucocorticoid excess. Notably, patients with CS, despite the long-term cure, may not regain their premorbid level of functioning and continue to experience persistent impairment of their quality of life and their cognitive function. These persistent symptoms following transient hypercortisolism are not well understood and highlight the importance of early detection and treatment.

## Data Availability

The datasets supporting the conclusions of this article are included within the article.

## Abbreviations

ACTH: Adrenocorticotropic hormone
BIPSS: Bilateral inferior petrosal sinus sampling
CD: Cushing’s disease
CS: Cushing’s syndrome
DHEAS: Dehydroepiandrosterone sulfate
eGFR: Estimated glomerular filtration rate
fMRI: Functional MRI
FSH: Follicle-stimulating hormone
GR: Glucocorticoid receptors
HIV: Human immunodeficiency virus
H-MRS: Magnetic resonance spectroscopy
HPA: Hypothalamus-pituitary-adrenal axis
IGF1: Insulin-like growth factor
LH: Luteinising hormone
MR: Mineralocorticoid receptors
MTL: Medial temporal lobe
TSH: Thyroid-stimulating hormone
